# The Relationship between NALP3 and Autoinflammatory Syndromes

**DOI:** 10.3390/ijms17050725

**Published:** 2016-05-13

**Authors:** Lorna Campbell, Irfan Raheem, Charles J. Malemud, Ali D. Askari

**Affiliations:** 1 Rheumatology Fellows at University Hospitals Case Medical Center, Cleveland, OH 44106-5076, USA; lorna.campbell@uhhospitals.org (L.C.); irfan.raheem@uhhospitals.org (I.R.); 2 Department of Medicine, Division of Rheumatic Diseases, Case Western Reserve University School of Medicine and University Hospitals Case Medical Center, 2061 Cornell Road, Cleveland, OH 44106-5076, USA; ali.askari@uhhospitals.org

**Keywords:** NALP3 inflammasome, interleukin IL-1β, autoinflammatory syndromes

## Abstract

The nucleotide-binding domain, leucine-rich repeat/pyrin domain-containing-3 (NALP3) inflammasome, which is required for synthesis of interleukin-1β, has been implicated in the pathogenesis of several autoinflammatory syndromes. This review of the literature summarizes the interconnectedness of NALP3 inflammasome with some of these disorders. Familial Mediterranean fever results from a mutation in the Mediterranean fever (MEFV) gene, which encodes the pyrin protein. Previous study results suggest that pyrin suppresses caspase-1 activation, perhaps by competing for the adaptor protein, termed, pyrin domain of apoptosis/speck-like protein containing a caspase-recruitment domain (ACS) which therefore interferes with NALP3 inflammasome activation. The nucleotide-binding domain, leucine-rich repeat/pyrin domain-containing-3 (NALP3) inflammasome is constitutively activated in cryopyrin-associated periodic syndromes due to gain-of-function mutations resulting from point mutations within the neuronal apoptosis inhibitor protein/class 2 transcription factor/heterokaryon incompatibility/telomerase-associated protein-1 (NACHT) domain of the NALP3 protein. Pyogenic arthritis, pyoderma gangrenosum and acne (PAPA) syndrome is caused by mutations in the genes encoding proline-serine-threonine phosphatase interacting protein 1 (PSTPIP1). These PSTPIP1 mutants are thought to bind to pyrin causing an increase in the pyrin domain of apoptosis/speck-like protein containing a caspase-recruitment domain (ASC) pyroptosome assembly leading to procaspase-1 recruitment and therefore its activation. Hyperimmunoglublinemia D syndrome is caused by mevalonate kinase (MVK) deficiency, which may be affected by protein accumulation that leads to NALP3 inflammasome activation. Tumor necrosis factor receptor–associated periodic syndrome is associated with mutations in the tumor necrosis factor receptor superfamily, member 1A (TNFRSF1A) gene which decreases the level of soluble tumor necrosis factor receptor-1 (TNFR1) leading to neutralization of tumor necrosis factor (TNF)-α. In general, these autoinflammatory disorders have shown a clinical response to interleukin-1 (IL-1) antagonists, suggesting that the NALP3 inflammasome serves a critical role in their pathogenesis.

## 1. Introduction

Because of the severity and the wide spectrum of their clinical manifestations, rare autoinflammatory syndromes have garnered significant research attention. Such syndromes include familial Mediterranean fever (FMF), cryopyrin-associated periodic syndrome (CAPS), pyogenic arthritis, pyoderma gangrenosum and acne (PAPA) syndrome, hyperimmunoglobulinemia D syndrome (HIDS), and tumor necrosis factor receptor-associated periodic syndrome (TRAPS) [1,2]. Because these disorders lead to recurrent and high levels of systemic inflammation, physicians have appropriately attempted to treat such conditions via anti-inflammatory drugs and immunosuppressive agents. IL-1 antagonism has demonstrated the greatest clinical efficacy for many of these disorders, suggesting that the NALP3 inflammasome, which is crucial for IL-1 activation, serves a key role in the pathogenesis of these conditions. By summarizing the basic immunology of the NALP3 inflammasome as well as the manifestations and treatments of these autoinflammatory syndromes, this review strives to elucidate the interconnectedness of the NALP3 inflammasome with the clinical manifestations of these syndromes and their response to immunosuppressive therapies.

## 2. Interleukin-1β

Interleukin-1β (IL-1β) is a potent pro-inflammatory cytokine that is responsible for multiple changes in immune system response [3]. In general, the IL-1 family of cytokines is closely associated with innate immunity. In that regard, as a member of the IL-1 family, IL-1β supports the host’s defense against infection. Thus, IL-1β produces an inflammatory response by increasing the expression of adhesion molecules on endothelial and mesenchymal cells and by inducing chemokine gene expression. These two actions promote the extravasation of immune cells from peripheral circulation into tissues. IL-1β also induces gene expression and synthesis of type 2 phospholipase A, cyclooxygenase-2 (COX-2), and inducible nitric oxide synthase (iNOS), leading to the production of platelet activating factor (PAF), prostaglandin E2 (PGE_2_) and nitric oxide (NO). The synthesis of these soluble mediators lowers the threshold to pain, produces fever and leads to vasodilatation and hypotension.

In addition, IL-1β serves as both a potent stimulator of IL-6 production as well as a growth factor for B-cell proliferation [3]. IL-1β also stimulates the generation of T_h_17 cells and acts as a costimulator for T-cell proliferation. In particular, IL-1β has received more attention than other members of the IL-1 superfamily because it plays an important role in the pathogenesis of several autoinflammatory disorders. Typically, the pathogenesis of these diseases involves an increase in the secretion of IL-1β, which is often related to an abnormal activation of the enzyme caspase-1. Additionally, several autoinflammatory diseases involve a mutation in the NALP3 inflammasome. Thus, the role of IL-1β and NALP3 inflammasome biology are currently under intense investigation to define essential targets to regulate autoinflammation.

## 3. NALP3 Inflammasome

Inflammasomes are multiprotein oligomers comprised of a sensor molecule known as a NOD-like receptor (NLR), which connects to caspase-1 through an adaptor protein called ASC [4]. ASC has two domains, which are called the pyrin domain, or PYD, and the caspase activation and recruitment domain, or CARD. ASC is encoded by PYCARD, which is universal to all inflammasomes. When the ASC pyrin domain interacts with the NLR, ASC then assembles into a large protein consisting of multimers of ASC dimers. Via its interaction with CARD, ASC then recruits pro-caspase 1 monomers into close proximity with one another. Inactive pro-caspase-1 then undergoes autocatalysis to form subunits p20 and p10, which then form an active heterodimer [5]. The NALP3 inflammasome, in particular, is composed of NALP3, or cryopyrin, as well as pro-caspase 1 and adaptor proteins, CARDINAL and ASC.

The NALP3 inflammasome can be efficiently stimulated by a wide variety of factors [6]. These include microbial molecules, known as pathogen-associated-molecular- patterns (PAMPS), including fungal zymosan and bacterial lipopolysaccharide. External ATP, a danger signal for the immune system, is also known to activate PAMPS as well. Specifically, it is hypothesized that the ATP-gated P2X7 receptor on the surface of antigen presenting cells requires hemichannel pannexin-1 to activate the inflammasome [7]. The bacterial toxin nigericin similarly activates the inflammasome via a pannexin-1 dependent process [8]. Other factors, such as microbial nucleic acids, muramyl dipeptide, toxins, asbestos, silica, alum as well as endogenous ATP stimuli, monosodium urate and calcium pyrophosphate crystals have all been implicated as inflammasome activators as well [5]. In summary, the NALP3 inflammasome is activated by a variety of factors, suggesting that it serves as a general detector of cell stress that results not only from pathogenic infection but also from intrinsic metabolic disturbances and trauma [2].

## 4. Functions of the NALP3 Inflammasome

Once NALP3 is activated, it fulfills a multitude of cellular functions. First and foremost, as previously discussed, it provides the signal for cleavage and therefore activation of IL-1β from pro-IL-1β. Moreover, a successful response to and control of viral, bacterial and fungal pathogens requires the inflammasome and IL-1β. Yet the NALP3 inflammasome is responsible not only for activation of appropriate inflammatory processes but also for the activation of pathological ones where excessive 1L-1β is produced [2]. For example, dysregulation of the inflammasome has been implicated in cancer. In hepatocellular carcinoma, in particular, all of the NALP3 components are downregulated or nonexistent according to study results reported by Wei *et al.* [9]. NALP3 plays a role in many inflammatory disorders as well. It leads to neuroinflammation seen in Alzheimer’s disease [10]. Because uric acid activates the NALP3 inflammasome, it has been implicated in the pathogenesis of gout [1,2]. Similarly, NALP3 is at least partially responsible for the hepatic inflammation seen in acetaminophen-induced liver injury [11] as well as in pronounced pulmonary inflammation following lung injury [12]. Interestingly, mutations in the NALP3 gene are associated with a group of autoinflammatory diseases called cryoprin-associated periodic syndrome, or CAPS [1,2].

## 5. The Role of Pyrin in the Pathogenesis of Familial Mediterranean Fever (FMF)

The main focus of this review is to examine the association between NALP3 and autoinflammatory disorders. The first of these is FMF. Almost all cases of FMF result from a mutation in the Mediterranean fever (MEFV) gene located on chromosome 16 [13]. The MEFV gene encodes the protein pyrin [14]. The MEFV gene mutated in FMF was identified by positional cloning [15]. Currently more than 50 FMF-associated mutations in MEFV have been found. Pyrin, the protein product of MEFV, is a 781-amino acid protein expressed in serosal and synovial fibroblasts, granulocytes, and cytokine-activated monocytes. Although the role of pyrin in IL-1 activation is controversial [14], it has been hypothesized that pyrin suppresses the activation of pro-caspase-1, perhaps by competing for ACS, and therefore pyrin interferes with NALP3 inflammasome activation. In support of this hypothesis, Chae *et al.* [15] revealed that the binding of pyrin constructs with FMF-associated mutations to caspase-1 was significantly reduced in comparison to the binding of wild-type pyrin to caspase-1. Of note, monocytes carrying the MEFV gene mutation have been shown to secrete increased levels of IL-1β [16]. Importantly, over-secretion of IL1β correlates with the number and penetrance of MEFV mutations. This finding suggested a dose effect for the number of MEFV mutations as it pertains to clinical presentation and disease penetrance. In summary, the NALP3 inflammasome is a core element of the pathology responsible for FMF.

Data for both the N-terminal and C-terminal end of pyrin has been elucidated [15]. The N terminus of pyrin, consisting of 90 amino acids, creates the PYRIN domain, which has a death-fold structure and allows for the interaction of pyrin with the adaptor protein ASC. ASC is known to interact with a NALP protein via its PYRIN domain and with procaspase-1 via CARD interactions. Thus, once the inflammasome complex consisting of ASC, NALP and pro-caspase-1 develops, two molecules of pro-caspase-1 are placed in close proximity, allowing for autocatalysis with the creation of the active catalytic p20 and p10 domains of caspase-1. Caspase-1 then cleaves pro-IL-1, a 31-kDa protein, into a 17-kDa structure. This is the biologically active form of IL-1 (*i.e.*, endogenous pyrogen) that causes fever and inflammation.

Although the role of pyrin in IL-1 regulation remains controversial, the study results reported by Chae *et al.* [15] also provided evidence that pyrin inhibited IL-1 activation. Thus, transfection of full-length mouse pyrin into a murine monocytic cell line suppressed IL-1 secretion. In these experiments, mouse ASC bound pyrin preferentially to caspase-1, suggesting that pyrin competed for ASC and therefore negatively regulated the NALP3 inflammasome. In contrast, other studies have suggested that pyrin activates pro-caspase-1 as well as IL-1 in human embryonic kidney cells [17]. Thus, pyrin may exhibit different effects on IL-1 activation and secretion in a cell-type-dependent fashion.

Of note, many of the FMF-associated pyrin mutations reside in the C terminal B30.2-domain of the protein [15]. However, the exact function of the pyrin B30.2 domain has not yet been elucidated. In an effort to better understand the function of the C-terminal domain, Chae *et al.* [15] used different glutathione-*S*-transferase-tagged (GST-tagged), domain-deleted versions of pyrin and myc-tagged caspase-1 to perform a GST-pull-down analysis. Although caspase-1 co-precipitated with pyrin deletion mutants at the N-terminus, much less caspase-1 was co-precipitated with the B30.2 domain deletion mutants. Additionally, the GST-tagged B30.2-domain alone strongly co-precipitated caspase-1. This finding suggested that the B30.2-domain is required and sufficient for the direct interaction of pyrin with caspase-1.

**Figure 1:**
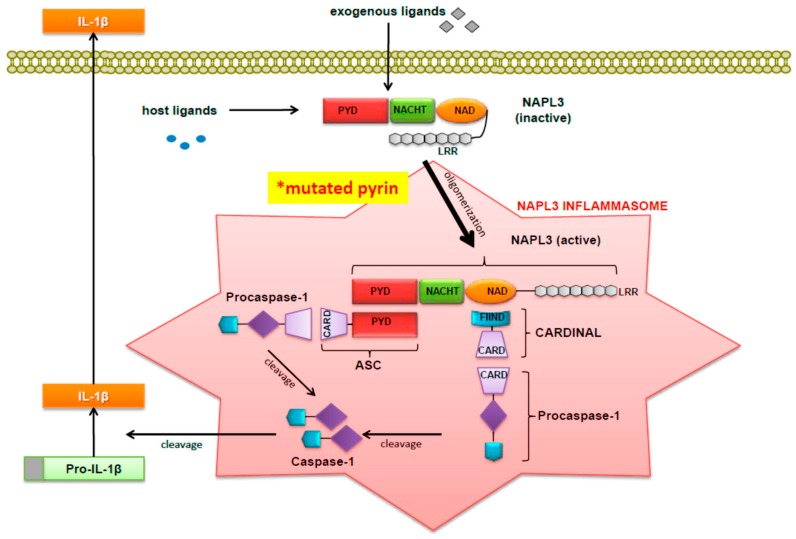
Activation of pro-IL-1β by the NALP3 Inflammasome. (Figure was originally published by Wang DQH *et al.* and cited in this paper with permission as reference [18]).

Because FMF-associated pyrin B30.2 mutants bound less caspase-1 than the wild type pyrin, Chae *et al.* [15] hypothesized that in FMF patients pyrin mutants have a reduced inhibitory effect on caspase-1 compared to its normal counterpart. Thus, in summary, there seems to be greater support for the hypothesis that pyrin suppresses IL-1 secretion. When examining the binding of caspase-1 to three common FMF-associated pyrin mutants, co-precipitation of caspase-1 was significantly decreased. In order to determine the effect of pyrin on the processing of IL-1, Chae *et al.* [15] then developed a construct that co-expressed caspase-1 and pro-IL1. This construct, called pIES-ICE IL1, was co-transfected into PT67 cells with an increasing amount of construct expressing full-length or B30.2 domain-deleted (NBC) pyrin. Both the full length and NBC pyrin suppressed IL-1 secretion, although NBC-pyrin suppressed IL-1 secretion significantly less and FMF-associated pyrin mutants had a reduced inhibitory effect on caspase-1 and therefore on IL-1 secretion. When considered together, these findings indicated that full-length pyrin had a direct inhibitory on effect on caspase-1. As shown in Figure 1 (which was originally published by Wang *et al.* [18]), the NALP3 inflammasome is depicted along with other related factors that have been implicated in the pathogenesis of FMF.

## 6. The Clinical Presentation of FMF

FMF is an autosomal recessive autoinflammatory disorder that is characterized by periodic episodes of fever, serositis, synovitis, and/or cutaneous inflammation [18]. These episodes exhibit varying levels of intensity and typically last between a few hours to 3 days. FMF is considered to be the most prevalent of all hereditary autoinflammatory disorders [19]. Approximately 150,000 people worldwide are estimated to have this condition, although its prevalence is not equal among ethnic groups [17]. As indicated by its name, FMF is more commonly found among ethnic groups originating from the Mediterranean littoral, including those of Arabic, Armenian, Jewish, North African and Turkish descent.

The pathogenesis of FMF appears to be complex in that more than 180 mutations in the MEFV gene have been implicated as a cause of FMF [20]. Pyrin containing FMF-associated mutations is thought to have less of an inhibitory effect on the inflammasome, leading to upregulated synthesis of IL-1β [18]. Therefore, inflammatory mechanisms trigger the disease, and marked acute-phase responses occur. These clinical responses include elevated white blood cell count, erythrocyte sedimentation rate, fibrinogen, C-reactive protein, and serum amyloid A (SAA) protein. These laboratory markers typically return to a normal baseline between attacks.

Interestingly, another laboratory finding that can be seen in FMF and detected via urinalysis is proteinuria occurring independently or in conjunction with microscopic hematuria [21]. During the intercritical period, patients may experience subclinical inflammation, allowing for the development of reactive amyloid A (AA) amyloidosis, the most severe complication of FMF. In that response, SAA has been identified as a major acute phase reactant. In fact, hepatocytes that produce SAA are transcriptionally regulated by pro-inflammatory cytokines [22]. Healthy individuals typically have a plasma SAA concentration of approximately 3 mg/L, whereas serum SAA levels can increase to greater than 2000 mg/L during an acute phase reaction. Importantly, AA amyloidosis can only develop when sustained overproduction of SAA occurs.

SAA protein can be cleaved, misfolded and assembled into an abnormal conformation known as a β-sheet, thus forming an amyloid fibril. These fibrils associate with other substances, including glycosaminoglycans and serum amyloid P, which creates the amyloid deposits that disrupt the structure and function of tissues and organs. The kidney typically is the first organ to be affected by these amyloid deposits [21].

However, patients with FMF can also develop proteinuria as a result of nonamyloidotic kidney disease. In that regard, Kukuy *et al.* [21] searched the registry of the Israeli national center for FMF, which included approximately 12,000 persons. They identified 25 patients whom underwent a kidney biopsy from 2001 to 2011 due to findings of proteinuria of at least 0.5 g/24 h. Based on their retrospective review of these biopsies, 40 percent of these patients had nonamyloidotic kidney disease. Of this cohort, 50% were diagnosed with focal glomerulonephritis and 10% were diagnosed with each of the following pathologies: focal segmental glomerular sclerosis, immunoglobulin A nephropathy, minimal change disease, focal glomerulonephritis with features of thrombotic microangiopathy, and focal interstitial nephritis. Interestingly, amyloidosis on biopsy was significantly associated with more severe FMF, which is understandable given the pathogenesis of secondary amyloidosis as described above.

FMF is typically a recurrent disease [18]. Approximately 90 percent of patients experience their first attack before the age of 20. These attacks are marked by symptoms similar to a traditional fever. However, the fever of FMF can be distinguished from a traditional fever by the onset of multiple clinical features. These features include peritonitis, arthritis and pleuritis, which occur with a corresponding prevalence rate of 95%, 50% and 40%, respectively. Less common features of FMF include pericarditis, scrotal swelling, myalgia, and erysipeloid erythema. Erysipeloid erythema has been postulated to occur in 7%–40% of FMF patients and consists of erythematous, shiny plaques that typically occur over the shins and have an erysipelas-like appearance [14]. Lastly, other less common symptoms of FMF include dizziness, an increase in appetite, change in taste sensation and even extreme emotional irritability.

## 7. Diagnosis of FMF

The diagnosis of FMF is made based on a combination of presenting symptoms and laboratory findings [14]. A criteria list used for the diagnosis of FMF consists of three main categories. These are major criteria, minor criteria, and supportive criteria. In order to obtain a diagnosis of FMF, a patient must have at least 1 major criterion, 2 minor criteria, 1 minor criterion with at least 5 supportive criteria, or 1 minor criterion with at least 4 of the first 5 supportive criteria. Three types of typical attacks constitute major criteria and include peritonitis (generalized), pleuritis (unilateral) or pericarditis, and monoarthritis of the knee, hip, and/or ankle. The minor criteria include incomplete attacks involving the abdomen, the chest and/or a joint, leg pain upon exertion and a favorable response to colchicine. Typical attacks are febrile, short in duration, and recurrent, meaning a patient has experienced at least 3 flares of the same symptoms. Short is defined as lasting between 12 h and 3 days. An incomplete attack is a painful and recurrent attack that differs from typical attacks in one or two features. These features include lack of fever, a duration that is longer or shorter than previously specified although no shorter than 6 h or longer than a week, lack of signs of peritonitis during abdominal attacks, localized abdominal attacks, and arthritis in joints not listed under the major criteria.

However, the list of supportive criteria is more extensive. For example, a family history of FMF, appropriate ethnic origin, and age less than 20 years at onset of disease are the first 3 criteria. The next 4 criteria refer to features of an attack, including severity and the requirement for bed rest, spontaneous remission, symptom-free interval and transient inflammatory response with one or more abnormal test results for leukocyte count, erythrocyte sedimentation rate, and elevated SAA, and/or fibrinogen. Episodic proteinuria and/or hematuria, unproductive laparotomy or removal of “white” appendix, and consanguinity of parents constitute the final 3 supportive criteria.

In addition to the aforementioned list of criteria for making a diagnosis of FMF, a diagnostic algorithm has also been established [14]. This algorithm includes 6 main levels, which are clinical findings, laboratory analysis, genetic analysis, diagnosis, therapy, and follow-up. A patient can be classified into having definite FMF *versus* possible FMF based on whether the individual experiences typical or incomplete attacks, demonstrates elevation of laboratory inflammatory markers, and carries a mutation of the MEFV gene. Patients experiencing typical attacks with abnormal laboratory findings do not require genetic analysis for making the definitive diagnosis of FMF. In contrast, a patient with incomplete attacks requires the demonstration of an associated abnormal genetic analysis in order to obtain a definitive diagnosis. Patients with incomplete attacks and unequivocal laboratory findings or genetic analysis carry a diagnosis of possible FMF. The diagnostic algorithm is helpful for clinical purposes. Patients labeled as having definite FMF should be prescribed colchicine and provided with appropriate follow-up whereas patients with possible FMF can be placed on a trial of colchicine or just be monitored on follow-up. Follow-up specifically involves assessing clinical response, proteinuria and SAA levels.

## 8. Treatment of FMF and Relationship to NALP3

Different treatments can be employed to combat the symptoms and effects of FMF. The most popular treatment for FMF is colchicine [14]. Colchicine typically is prescribed at a dosage range of 1–2 mg/daily. The majority of patients with FMF are asymptomatic with use of this regimen. However, approximately 10% of FMF patients do not respond to colchicine and continue to experience persistent symptoms. When this occurs, it is recommended that physicians increase the dosage by 0.5 up to 2 mg/day until an effective dose is reached. Based on FMF management guidelines published by French and Israeli experts, 3 months of a steady colchicine dosage is advised prior to increasing the dose [23]. The recommended maximum doses are 3 mg/day in adults and 2 mg/day in children.

Colchicine has different mechanisms of action based on its concentration. Nuki reviewed the literature on the mechanisms attributed to colchicine at various concentrations in reference to the treatment of gout [24]. Given that the end result of the action of colchicine is to reduce inflammation, it is likely that colchicine works in a similar fashion in the treatment of FMF. Thus, Nuki [24] indicated that colchicine at micromolar concentrations suppressed monosodium urate crystal-induced protein-3 NALP inflammasome-driven caspase-1 activation, IL-1β processing and IL-1β release. Additionally, l-selectin expression by neutrophils was also suppressed. However, at nanomolar concentrations, colchicine inhibits the release of a crystal-derived chemotactic factor from neutrophil lysosomes. In addition, at these concentrations colchicine changes the distribution of adhesion molecules on endothelial cells, thus impairing neutrophil adhesion to the endothelium. Lastly, colchicine inhibits the generation of superoxide anions from neutrophils, which typically is induced by monosodium urate crystals. Interestingly, in addition to treating the inflammatory components of FMF, colchicine also stabilizes the quantity of proteinuria in patients with amyloid nephropathy, which can lead to the resolution of renal disease in those patients with a creatinine level below 1.5 mg/dL.

However, colchicine is known to cause gastrointestinal side-effects which generally consist of nausea, vomiting and diarrhea [24]. In addition, based on data reported by Cerquaglia *et al.* [25] regarding the use of colchicine in the treatment of FMF, colchicine was shown to result in complete remission in 5% of patients, strong improvement in 80%, and no response or intolerance in 10%–15% of patients with FMF. With respect to the gastrointestinal side-effects of colchicine, it was hypothesized that colchicine may affect the gastrointestinal mucosa via 2 different mechanisms; inhibition of the Na^+^ K^+^ exchange pump which regulates the transport of water and electrolytes as well as by direct mucosal damage of the small and large bowel.

**Figure 2:**
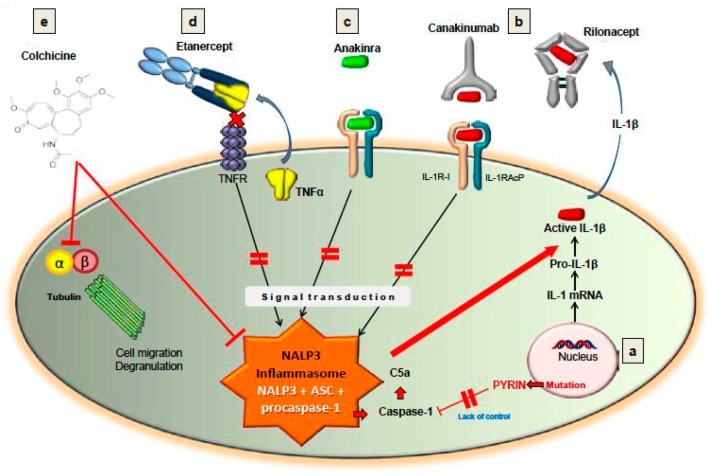
Treatment with cochicine, TNF blockade (e.g., Etanercept) or IL-1β blockade (e.g., Anakinra, Canakinumab, Rilonacept) can lead to inactivation of the NALP3 inflammasome (Figure was originally published by Wang DQH *et al.* and cited with permission in this paper as reference [18]). Abbreviation: C5a, Complement protein fragment C5a.

Importantly, there are treatment options for FMF patients who do not respond to colchicine [23]. There are inhibitors of TNF-α, including etanercept and infliximab, as well as the IL-1 receptor antagonists, anakinra, canakinumab and rilonacept. Of note, anakinra, canakinumab and rilonacept impede the activity of the NALP3 inflammasome by antagonizing IL-1. Case series have reported success with both anakinra and canakinumab for FMF patients [26,27]. In addition, the clinical efficacy of rilonacept was assessed in a randomized controlled study of 14 colchicine-resistant FMF patients [22]. In that clinical trial, 8 FMF patients successfully responded to rilonacept. Two of the non-responders were found to carry unusual heterozygous mutations in the MEFV gene. The number of attacks was reduced with rilonacept therapy from 3.3 attacks per month to 0.77 per month compared to 2.0 per month with placebo. Importantly, no significant adverse effects were reported. Of note, Israeli and French experts who had published guidelines for FMF treatment recommended that colchicine-resistant patients begin anti-IL-1 therapy [28]. They advised to initially use a short half-life molecule, such as anakinra, in order to determine its efficacy in the individual patient prior to employing a long half-life IL-1 targeted therapy [23]. In summary, Figure 2, (originally published by Wang *et al.* [18]), demonstrates the sites of action for several treatment modalities for FMF and their association with the NALP3 inflammasome.

## 9. Cryopyrin Associated Periodic Syndrome (CAPS)

A group of inflammatory disorders called cryopyrin-associated periodic syndromes or CAPS results from mutations associated in the NALP3 inflammasome [1]. CAPS encompass three distinct autoinflammatory conditions, including familial cold autoinflammatory syndrome (FCAS), Muckle-Wells syndrome (MWS) and neonatal onset multi-systemic inflammatory disease/chronic infantile neurological cutaneous articular syndrome (NOMID/CINCA). Their respective names originate from the term cryopyrin, the original term for the gene encoding Nod-like receptor protein 3 (NLRP3). These conditions form a spectrum of diseases of increasing severity, with the mildest, intermediate and most severe manifestations represented respectively by FCAS, MCW and NOMID/CINCA. The symptoms of CAPS include recurrent fevers, urticarial-like rashes, arthralgias and/or arthritis, amyloidosis, sensorineural hearing loss, and eye problems. In the case of NOMID/CINCA, patients can experience neurological difficulties as well. These include severe headaches, increased intracranial pressure, chronic aseptic meningitis and even mental retardation.

Cold is known to trigger febrile episodes in FCAS including cold-induced urticaria [29]. Thus, the “cold trigger” associated with FCAS is an autosomal condition that is inherited from one affected parent. In fact, the “cold trigger” is one of the unique features of FCAS. However, it has also been noted that FCAS, NOMID/CINCA and MWS are syndromes, which clinically are closely related to one another [30]. Thus, it was reported that clinical responses in families and individuals diagnosed with MWS triggered by exposure to cold as one feature of FCAS also presented with mild features of NOMID/CINCA [30]. However, a major difference between the clinical presentations of FCAS, MWS and NOMID was the finding of meningitis. Thus, in NOMID, meningitis was a prominent feature, whereas in FCAS and MWS meningitis was absent, but headache prominent in MWS [31].

## 10. CAPS and the NALP3 Inflammasome

The NALP3 inflammasome is constitutively activated in CAPS due to gain-of-function mutations resulting from point mutations within the NACHT domain of the NALP3 protein. Researchers have shown that monocytes and macrophages from MWS patients release basal levels of IL-1β even in the absence of an external stimulus [29]. Thus, Agostini *et al.* [32] isolated monocytes from a patient with Muckle-Wells syndrome who possessed the NALP3 R260W mutation. These monocytes were stimulated with LPS and the level of IL-1β found in the culture supernates was then measured. Interestingly, IL-1β was detectable in supernates prior to monocyte simulation. After a 24-h stimulation with LPS, IL-1β levels increased as expected but by comparison, the supernates from the macrophages isolated from a normal donor did not have any detectable IL-1β prior to stimulation with LPS. Following the identical stimulation with LPS for 24 h, these macrophages produced only slightly higher levels of IL-1β than the pre-stimulated MWS monocytes. Not surprisingly, TNF levels increased with LPS stimulation as well. However, TNF release, which is not caspase-dependent, was slow and not detectable after 4 h, whereas the secretion of IL-1β was rapid and noticeable after just 2 h. Therefore, the overall increased cytokine production observed within MWS and other CAPS likely results from increased release of IL-1β.

## 11. Treatment of CAPS

With such persuasive evidence implicating the NALP3 inflammasome and IL-1β in the pathogenesis of CAPS, treatment with IL-1 receptor antagonists was considered as a way to ameliorate symptoms [2]. In that regard, anakinra, was shown to effectively treat all three forms of CAPS. Goldbach-Mansky *et al.* [33] published the results of a study in which 18 patients with the diagnosis of NOMID or CINCA ranging in age from 4 to 32 were treated with anakinra. Anakinra was initiated at a dose of 1 mg/kg/day by subcutaneous administration, which was increased to a maximum of 2 mg/kg/day if laboratory or clinical measures suggested continued disease progression. Efficacy was assessed after 1, 3 and 6 months. Patients with a treatment response underwent an inpatient withdrawal period. In that case, anakinra was restarted if a disease flare occurred so that patients were monitored for an additional 24 months. Of note, all 18 study participants had active disease upon entry into the study, including but not limited to laboratory findings of elevated acute phase reactants despite treatment with immunomodulatory medications and corticosteroids.

The overall response to anakinra was impressive. In fact, all 18 patients had an immediate clinical response. Conjunctivitis and rash resolved within 3 days in all cases. Subjective diary scores significantly decreased at 3 months and all patients had a significant decrease in levels of SAA, CRP and ESR. After 3 months of treatment with anakinra, 11 patients were placed on a withdrawal period for a maximum of 7 days with 10 out of the 11 patients experiencing a flare of the disease. Thus, as a result of the severity of some of these flares, the withdrawal period was not attempted for the rest of the study participants. Upon the resumption of anakinra, patients demonstrated a quick response that was sustained at the 6-month follow-up period. At 6 months, 6 patients demonstrated improved hearing based on audiography and 9 patients had stable hearing. One patient had improved hearing at high frequencies but experienced a worsening of hearing at low frequencies. All patients demonstrated stable vision. Pain, global assessments by parents and physicians, and scores on the Childhood Health Assessment Questionnaire were significantly improved. At 3 and 6 months, the median prednisone dose decreased significantly in comparison to baseline. Complete remission of inflammatory symptoms was experienced by 8 of 18 patients at 3 months and by 10 of 18 patients at 6 months. Finally, patients also demonstrated improvement in neurologic symptoms as well.

Unfortunately, patient tolerance to anakinra may be low due to side-effects involving major injection site reactions, upper respiratory tract infections and other general infections [2]. Fortunately, 2 additional IL-1 antagonists have been approved for patients with CAPS [2]. These are associated with less severe injection site reactions and a different side-effect profile.

Rilonacept, also known as arcalyst, is a fusion protein between IL-1R and IL-1Ra. It specifically targets IL-1β and is approved for treatment of both FCAS and MWS in patients who are 12 years of age or older. However, rilonacept can cause minor injection site reactions, upper respiratory infections as well as other infections. Canakinumab is a humanized monoclonal antibody that neutralizes the biological activity of IL-1β and is approved for both FCAS and MWS patients above 4 years of age. However, canakinumab, also known as ilaris, is associated with upper respiratory infections and vertigo, but only negligible injection site reactions.

Importantly, persuasive concrete data has now emerged which demonstrated the clinical efficacy of these anti-IL-1 agents for treating autoinflammatory syndromes. In fact, open label extension and double-blind placebo-controlled randomized clinical trials showed that IL-1 blockade with anakinra, rilonocept or canakinumab have clinical efficacy for treating these disorders. Although it had been known that the mechanism of action for rilonocept involved the binding to and neutralization of the IL-1 receptor, additional data showed that rilonacept bound both IL-1α and IL-1β with high affinity. In this regard, Dhimolea [34] suggested that rilonacept might have a better inhibitory profile *in vivo* when compared to the other IL-1 blockers. Conversely, canakinumab, a human anti-IL-1β monoclonal antibody, which only neutralizes IL-1β signaling was shown to suppress inflammation. Of note, canakinumab was also reported to have significant clinical benefits over other competitive therapies for the treatment of cryopyrin-associated periodic syndromes, including bimonthly treatments and its approved use in children [34].

## 12. Pyogenic Arthritis, Pyoderma Gangrenosum and Acne (PAPA) Syndrome

PAPA Syndrome is characterized by early onset of recurrent flares of pyogenic yet sterile arthritis in individuals demonstrating an autosomal dominant inheritance pattern [35]. These symptoms can also be associated with a variety of skin manifestations including, ulcerations, pyoderma gangrenosum and severe cystic acne. Laboratory findings are consistent with systemic inflammation but do not provide enough information to make a definitive diagnosis. Importantly, patients with PAPA syndrome have persistent symptoms that progress into adulthood and lead to significant joint destruction if not treated effectively. The first case of PAPA syndrome was reported in 1975 [36]. It first was described as a heritable disease in 1997 based on findings of similar symptomatology within an extended family [35].

PAPA syndrome is not caused by mutations of the NLRP3 *per se*, such as is seen with CAPS. Rather PAPA syndrome is caused by mutations in the genes encoding proline serine threonine phosphatase-interacting protein-1 (PSTPIP1). However, the mechanism by which PSTPIP1 is thought to lead to increased inflammation remains a topic of debate. PSTPIP1 is a cytoskeletal adaptor protein that interacts with actin. Yet it also interacts with pyrin. Two missense mutations in PSTPIP1 have been associated with PAPA syndrome [16]. These mutations are clustered in the protein’s coiled-coil region. These mutations interfere with the binding of PEST-type protein tyrosine phosphatase PTP-PEST to PSTPIP1, leading to hyperphosphorylated variants of PSTPIP1. Shoham *et al.* [37] revealed that these variants have a higher affinity for pyrin than the non-mutated forms. They also demonstrated that not only are these mutations associated with overproduction of IL-1β, but LPS-stimulated monocytes isolated from a PAPA syndrome patient have increased levels of IL-1β as well. According to these findings, PAPA-associated PSTPIP1 mutants were identified as having an inhibitory effect on pyrin, leading to increased IL-1β production, given that the pyrin was thought at that time to exhibit an inhibitory effect on the NALP3 inflammasome.

However, Yu *et al.* [17] suggested a different mechanism of action. They indicated that the PSTPIP1 mutants bound to pyrin and as a result caused an increased ability to assemble the ASC pyroptosome instead of inhibiting its anti-inflammatory activity. They elucidated this mechanism by studying the effect of retrovirus-mediated transient expression of PAPA-associated PSTPIP1 mutants in THP-1 monocytes and its effects on pro-caspase-1 activation and generation of IL-1β. Their results revealed that pyrin as well as PSTPIP1 were homotrimers and that pyrin served as a receptor for PSTPIP1 within the cytoplasm. The pyrin homotrimer had a B-box that covered a region known as the PYD, thus inhibiting pyrin from being fully active. Thus, PSTPIP1 binds to the B-box of pyrin and then uncovers the PYD region, allowing ASC to interact with the PYD region and oligomerize to form the ASC pyroptosome. Lastly, pro-caspase-1 is recruited and activated. Therefore, pyrin was shown to be an activator of the ASC pyroptosome, and PSTPIP1 serves an important role in the activation of the pyroptosome.

## 13. Treatment of PAPA Syndrome

According to case reports, several therapies have been successfully utilized to treat patients with PAPA syndrome. These medications include corticosteroids, azathioprine, sulfasalazine, leflunomide, mycophenolic acid, TNF-α inhibitors and IL-1 antagonists [38]. Given that the pathogenesis of PAPA syndrome is related so closely to the NALP3 inflammasome, it is understandable that IL-1 antagonists have effectively treated some patients with PAPA syndrome [39]. Brenner *et al.* [40] as well as Dierelhuis *et al.* [41] each reported a case of successful treatment of PAPA syndrome with anakinra. In addition, because IL-1β potently induced TNF-α in these patients, TNF-α blockade has also been used successfully to treat PAPA syndrome [35]. Based on the published data, it appears that TNF-α inhibitors rather than IL-1 antagonists have demonstrated the most consistent clinical response [39]. However, clinical responsiveness varies from patient to patient. Demidowich *et al.* [39] assessed the effectiveness of different therapies in 5 patients who possessed both PSTPIP1 mutations as well as the classic clinical symptoms of PAPA syndrome. Three patients were treated with infliximab. Of these, two patients demonstrated a good clinical response, although one of them had to discontinue the medication secondary to a negative side effect. A different TNF-α inhibitor, adalimumab, effectively treated both of the patients whereas anakinra had conflicting effects in the four patients who received it. One patient demonstrated a good clinical response, two patients demonstrated inadequate responses, and the fourth patient discontinued the medication because of adverse side- effects.

In summary, multiple immunosuppressive agents have been used in the treatment of PAPA syndrome. However, therapy with biologic drugs has not been effective in all cases. Smith *et al.* [30] commented that cystic acne seemed to be least responsive to therapy compared to severe pyoderma gangrenosum, suggesting that differing disease mechanisms may have important implications in pathogenesis. Thus, the differing response to TNF-α inhibition and IL-1 antagonism amongst patients with PAPA syndrome may also be a reflection of the function(s) of PSTPIP1. In that regard, PSTPIP1 is likely to affect multiple biochemical pathways in many immune-related cells, including T-cells, neutrophils, monocyte-derived cells and NK cells.

## 14. Other Autoinflammatory Syndromes

Other autoinflammatory syndromes have been deemed to have an inflammasome-based etiology primarily due to the effectiveness of IL-1 antagonist therapy in treating them [1]. Although it is hypothesized that their etiologies are related to the NALP3 inflammasome, the exact mechanism for each of these diseases has not been determined. These syndromes include HIDS with fever syndrome, TNF receptor-associated periodic syndrome, systemic juvenile idiopathic arthritis, adult-onset Still’s disease, relapsing polychondritis, Schnitzler’s syndrome, Sweet’s syndrome, Behcet’s disease, and anti-synthetase syndrome. Given that mutations have been identified for both HIDS with fever syndrome and TNF receptor-associated periodic syndrome, these will be addressed in the last section of this review.

## 15. Hyperimmunoglobulinemia D Syndrome (HIDS)

HIDS is a very rare autoinflammatory disorder in that only 180 patients had been identified worldwide as of 2001 [41]. HIDS consists of recurrent febrile episodes that begin in infancy and have associated with it, lymphadenopathy, arthralgias, rashes and gastrointestinal difficulties. As adults, a small percentage of patients also experience neurologic symptoms, which can include mental retardation, ataxia, seizures and ocular problems. The fever typically lasts for 4–6 days and can be provoked by a wide number of factors, including vaccination, surgery, minor trauma or other stressors. The most common accompanying symptoms are abdominal pain, vomiting, diarrhea and cervical lymphadenopathy. Patients may also present with hepatosplenomegaly, headache, arthralgias, rashes as well as oral and vaginal apthous ulcers. These symptoms disappear once the fever abates, although skin and joint manifestation can take longer to resolve.

Patients are diagnosed with HIDS based on the detection of an increase in the excretion of urinary mevalonic acid accompanied by elevated levels of serum immunoglobulin D [42]. With the exception of some patients less than 3 years of age, HIDS patients exhibit continuously elevated levels of IgD, defined as levels above 100 IU/mL. The majority of patients also exhibit elevated levels of IgA. During febrile attacks, patients are found to have significant elevations of urinary mevalonic acid levels, whereas mevalonic acid levels may be only minimally elevated in between episodes. The diagnosis can be confirmed by identifying 2 disease-causing mutations in the mevalonate kinase (MVK) gene or demonstration of reduced MVK enzyme activity.

HIDS is caused by mutations in the MVK gene [43]. MVK is an important early enzyme in isoprenoid and sterol synthesis [43]. Most HIDS patients are heterozygous for missense mutations in the MVK gene [39]. Thus, Cuisset *et al.* [43] identified mutations responsible for HIDS within the MVK gene. In that study, the most common missense mutation, V337I occurred in 80% of the HIDS patients. V337I is located on exon 10 where the nucleotide guanine replaces adenine. Importantly, the V337I mutation changes the folding of the MVK protein it encodes, thus altering its structural configuration to make the expression of MVK *in vivo* temperature-sensitive [43]. As a result, the V377I mutation is associated with a deficiency of MVK [43]. However, generally, HIDS patients have approximately 5%–15% residual MVK enzyme activity [42]. Importantly, patients with other MVK mutations which result in no residual MVK activity are deemed to have MVA, which presents clinically with more severe symptoms.

Celsi *et al.* [44] demonstrated that there does not appear to be a direct relationship between MVK and the NALP3 inflammasome, although they suggested that an indirect relationship might exist. Thus, Celsi *et al.* [44] created a murine cellular model that closely approximates the pathogenic conditions of HIDS by creating a knockdown of mevalonate kinase in a murine microglial cellular model. These cells, referred to as BV-2 cells, did not have an increased expression of NALP3. Interestingly, they also researched the effect of lovastatin treatment on MVK expression in these BV-2 cells. MVK protein expression increased with stimulation from lovastatin alone and with stimulation of lovastatin plus LPS from *E. Coli* 055:B5. However, NALP3 expression was only increased with combined treatment of lovastatin and LPS. Interestingly, lovastatin treatment resulted in a global increase of MVK protein content that was up to 15 times compared to untreated cells. Therefore, it is postulated that MVK deficiency could be affected by protein accumulation that leads to NALP3 activation.

## 16. Treatment of HIDS

Treatment of patients with HIDS has proven to be difficult. Many standard anti-inflammatory drugs, such as colchicine, non-steroidal anti-inflammatory drugs and steroids, are not effective therapeutic agents [38]. However, patients with HIDS have demonstrated positive responses to other agents as well. Thus, one of the IL-1 antagonists, canakinumab, has effectively treated HIDS [45] and according to Arostegui *et al.* [46], canakinumab can markedly reduce the frequency of acute flares in patients with active HIDS. Interestingly, HIDS tends to be unresponsive to steroids and only partially responsive to NSAIDs [47]. In fact, Arkwright *et al.* [47] reported the case of a patient with HIDS who responded positively to etanercept where the patient’s symptoms completely resolved during the first 3 months of therapy although the effect of etanercept appeared to wane over time. There have been other reports of a few HIDS patients who have also positively responded to etanercept [23]. Lastly, IL-6 blockade has been attempted for treatment of HIDS. One case report identified tocilizumab as successfully treating a young female with HIDS who had been treated with multiple other agents without success [48].

Simvastatin was proven to reduce the excretion of mevalonic acid as well as reducing the number of febrile episodes for most HIDS patients. Unfortunately, this difference was later determined to be only statistically significant, since the clinical use of simvastatin led to disappointing clinical results [45]. Fortunately, anakinra has been reported to be effective in several case reports. In that regard, Bodar *et al.* [45] conducted a prospective observational study in order to determine the effectiveness of anakinra for remission of mevalonic aciduria (MA) and HIDS. Patients with MA received daily treatments with anakinra, whereas patients with HIDS could choose between daily treatments or on-demand treatments for 4–7 consecutive days during an attack. Children less than 16 years of age were started on a dose of 1 mg/kg/day but could receive a dose of up to 2 mg/kg/day. Adults were given doses between 100 and 200 mg/day. Patients or their parents completed daily score cards to assess disease activity and to calculate a “clinical score”. A fever attack was defined as having a temperature of 38.0 degrees Celsius or higher with a “clinical score” of at least 20. Serum was collected during the study to assess cytokine levels. Eleven of the twelve invited patients participated in the study. On-demand treatment resulted in a good clinical response in eight of twelve observed fever episodes in patients with HIDS, meaning there was at least a 50% reduction in duration of fever. In contrast, continuous anakinra treatment in two children with classic MA was not as favorable, and a higher dose of anakinra may be more effective for MA patients. Specifically, one patient experienced a clinical response within 24 h but then had two additional febrile attacks in the next 19 weeks with partial remission of one of these attacks with increase in the dose of anakinra to 2 mg/kg. The second MA patient did not experience remission of symptoms despite 4 months of therapy.

## 17. TNF Receptor-Associated Periodic Syndrome (TRAPS)

TRAPS is an autoinflmmatory disorder characterized by prolonged febrile episodes with cutaneous, localized inflammation [49]. TRAPS is inherited in a dominant fashion. TRAPS used to be called familial Hibernian fever because it originally was described in 1982 amongst an Irish family of 16 members who had experienced symptoms of episodic fevers, painful myalgias and painful erythema [44]. Many patients experienced abdominal pain as well. Interestingly, patients have been identified from a wide range of racial and ethnic backgrounds, including individuals of Scottish, Finnish, Dutch, Puerto Rican, and mixed Irish, English and German descent [49]. These patients experienced febrile attacks that lasted for more than a week which was non-responsive to colchicine.

## 18. Pathogenesis of TRAPS

Researchers have determined that many TRAPS patients had mutations in tumor necrosis factor receptor superfamily, member 1A (TNFRSF1A), the gene that encodes the p55 TNF receptor type I (TNFR1) [50]. McDermott *et al.* [51] discovered six variable missense mutations in seven affected families in the TNFRSF1A gene. Five of the six mutations were thought to disrupt extracellular disulfide bonds [50]. These patients were found to have only half of the normal levels of soluble plasma TNFRSF1A levels. They were also found to have leukocytes with a C52F mutation with reduced clearance of the p55 TNF receptor from the surface of activated cells. Therefore, less p55 was released into the supernates of cultured cells. In summary, it was hypothesized that an impaired ability to down-regulate membrane TNFRSF1A with a reduced shedding of soluble receptor leads to TRAPS.

Specifically, TNF homotrimers induce signaling by grouping together onto membrane receptors. Cleavage of the cysteine-rich extracellular domains of TNFR1 by metalloproteases likely assists clearance of TNFRSF1A from the plasma membrane. TNFR1 shedding then creates a group of soluble receptors that compete with the membrane form of TNFR1. In other words, the role of the non-mutated TNFR1 is to release TNFR1 from the plasma membrane, which can then act to neutralize TNF-α activity [52]. In the event that mutations in the TNFRSF1A gene occur, neutralization of TNF-α by soluble TNFR1 does not occur. As a result, symptoms and signs of increased TNF-α-mediated inflammation develops, which can then lead to TRAPS.

Of note, TRAPS appears to be closely associated with the activity of the NALP3 inflammasome. Alvarez and Munoz-Fernandez [53] studied caspase-1 activity in the human neuroblastoma cell lines, SK-N-MC cells, and showed a two-fold increase in caspase-1 activation after treating these cells with 20 ng/mL of TNF-α when compared to untreated cells. This increase in caspase-1 activation was even higher with increasing concentrations of TNF-α. Because TNF-α has been implicated in activating caspase-1 activation and in causing TRAPS, the NALP3 inflammasome likely plays a central role in the pathogenesis of TRAPS.

Importantly, TNFRSF1A is expressed in a wide variety of cells and is responsible for multiple biological actions [50]. These responses include increased expression of adhesion molecules, induction of cytokine secretion, leukocyte activation, intracellular pathogen resistance, angiogenesis, anemia, fever and cachexia. Therefore, the clinical manifestations of TRAPS are multifold. To address this issue, Toro *et al.* [49] conducted a review of the clinical manifestations of TRAPS manifested by 25 patients admitted to the National Institutes of Health Clinical Center in Bethesda, Maryland. All patients had mutations in exons 2 through 4 of TNFRSF1A which affected the p55 TNF receptor extracellular domain. One-hundred percent of these patients experienced fever, 88% had abdominal pain, and 80% complained of myalgia. Abdominal pain at times was severe. In fact, four patients were hospitalized for emergency laparotomies due to the severity of pain. Additional clinical findings with associated prevalence include headache (68%), arthralgia (52%), pleuritic chest pain (40%) and amyloidosis (8%). Cutaneous manifestations included erythematous patches (84%), erythematous plaques (40%), ecchymotic lesions (36%), and conjunctivitis with or without periorbital edema (44%). In total, 84% of patients with TRAPS experienced at least one cutaneous symptom during flares. Ten patients underwent a skin biopsy. A review of their biopsy specimens revealed a normal epidermis with superficial and deep interstitial and perivascular inflammatory infiltrates of monocytes and CD3^+^ T-cells. Many of these cells were also positive for CD4, CD8 and CD68. Of note, the different types of skin lesions demonstrated the same immunohistological characteristics.

## 19. Treatment of TRAPS

Episodes of fever associated with TRAPS respond to corticosteroids [54]. Unfortunately, some patients require increasingly higher doses or repeated doses of steroids throughout the course of their disease, leading physicians to seek additional therapies. Because TRAPS is caused by the impaired removal of TNFR1 from the plasma membrane, anti-TNF therapy has been assessed for the treatment of some TRAPS patients. Specifically, etanercept has successfully treated some patients with TRAPS. In that regard, Drewe *et al.* [55] reported the response of seven TRAPS patients who received subcutaneous etanercept at a dose of 25 mg twice weekly for 24 weeks. Five patients demonstrated improvement in subjective symptoms and CRP levels coupled to a reduction in the amount of corticosteroids [56]. However, one of these five patients later became unresponsive to etanercept following a 3-month washout period.

Bulua *et al.* [57] conducted a prospective, open-label, dose-escalation study of etanercept in the treatment of fifteen patients with TRAPS. In this study, etanercept reduced the symptoms and laboratory levels of inflammatory markers in the TRAPS patients in a dose-dependent fashion. However, etanercept did not completely normalize acute phase reactants nor did it fully resolve clinical symptoms. After following these patients for 10 years, it was discovered that many of them had been changed to an IL-1β receptor therapy or had discontinued biologics altogether, most often due to lack of efficacy or injection site reactions. These latter patients had received etanercept for a median of 3.3 years, yet those who had continuous therapy noted a reduction in symptoms at follow-up.

Although etanercept has treated some TRAPS patients effectively, infliximab often is ineffective in treatment of TRAPS and may even cause paradoxical episodes of severe inflammation [53]. In order to understand the difference between the response of these patients to etanercept and infliximab, Nedjai *et al.* [58] obtained peripheral blood mononuclear cells from nine patients with TRAPS as well as from normal controls. The T50M mutation in TNFRSF1A was found in all nine of the patients, who belonged to the same family. Caspase-3 activity, NF-κB subunit activity, and cytokine secretion was measured in these cells. Whereas the normal controls demonstrated enhanced caspase-3 activity as a result of administration of infliximab, the nine patients with TRAPS did not demonstrate pro-apoptotic induction of caspase-3 activity. Instead, these cells showed an increased activity of the anti-apoptotic c-Rel subunit activity along with impressive increases in the secretion of pro-inflammatory cytokines, including IL-1β, IL-6, IL-8, IL-12 as well as IL-1 receptor.

Typically, when TNF binds to TNFR1 on the surface of immune cells, the TNF/TNFR1 complex is internalized, pro-apoptotic proteins are recruited which initiates the caspase cascade. The results of studies have shown that in TRAPS patients with cysteine or threonine mutations in the extracellular cysteine-rich domain of TNFR1, TNF does not initiate apoptosis. Thus, changes to the conformation of the extracellular region of the TNFR1 impede receptor internalization and subsequent activation of apoptotic pathways. In that regard, Nedjai *et al.* [58] showed that patients carrying the T50M mutation not only failed to cause internalization of TNFR1, but also activated and up-regulated antiapoptotic c-Rel subunits as well. In fact, previous studies had shown that up-regulation of c-Rel also led to manganese superoxide dismutase up-regulation, which causes resistance to induction of apoptosis [58]. In addition, the results from other studies have determined that IL-12 transcription by macrophages is mediated by c-Rel [59]. Therefore, it is not surprising that the T50M mutation, which up-regulates the c-Rel subunit, also increased the secretion of pro-inflammatory cytokines in this study [60].

Although infliximab and etanercept both bind to TNF, infliximab creates a more stable complex with soluble TNF and also binds more avidly to transmembrane TNF when compared to etanercept. In addition, the other TNF receptor, TNF2, has high rates of dissociation with TNF. Thus, Nedjai *et al.* [58] hypothesized that because etanercept binds more transiently to TNF, it likely has a different binding specificity for the two TNF receptors, thus accounting for the different responses to these two agents in patients with TRAPS. In conclusion, due to the findings of an increased level of inflammation in response to infliximab in some patients with TRAPS, the use of infliximab is strongly advised against for the treatment of TRAPS [54].

As previously indicated, anti-IL 1 therapy has been successfully used for treatment of TRAPS. In support of this, Gattorno *et al.* [61] assessed the effectiveness and safety of anakinra in patients with TRAPS who required high cumulative doses of steroids. These investigators completed a cohort study of five patients; four patients were less than 18 years of age at the time of study enrollment. Three of the children had frequent and prolonged attacks whereas one child and the adult had a chronic course with fluctuating yet persistent symptoms. Patients received anakinra at a dose of 1.5 mg/kg/day. Interestingly, all patients responded quickly to anakinra and experienced resolution of symptoms with normalization of acute-phase reactant levels. During the follow-up period, which was 11.4 months on average for this cohort, patients did not develop a fever or other symptoms of active disease. The levels of the acute phase reactants remained normal as well. Importantly, this study did not identify any severe infections or major adverse reactions. In summary, continuous treatment with anakinra seems to be an effective and safe management strategy for patient with TRAPS who demonstrate more recurrent or prolonged flares.

## 20. Conclusions and Future Perspectives

This review focused on the underlying molecular mechanisms, clinical presentation and treatment of FMF, CAPS, PAPA syndrome, HIDS, and TRAPS. For the most part, these disorders have demonstrated a good response to IL-1 antagonists, suggesting that the NALP3 inflammasome serves a critical role in the pathogenesis of these autoinflammatory disorders. Instead of targeting downstream effects of the NALP3 inflammasome, such as IL-1, future development of pharmaceutical drugs for treating these disorders may actually be preventative by blocking pathologic inflammasome stimulation, thus interfering with the activation of caspase-1 and formation of activated IL-1. Because the NALP3 inflammasome has been implicated in the pathogenesis of more common disorders as well, research into the development of such pharmaceutical drugs and into the series of events surrounding NALP3 inflammasome activation would provide great benefit to society.

Finally, it will also be important to consider that, in addition to the role the inflammasome plays as an activator of IL-1 [1,2], inflammasome activation also causes pro-IL-18 to be activated as well [61,62,63]. In that respect, IL-18 is one of 9 members of the IL-1 superfamily and IL-18 and IL-1β are closely related in structure [63]. Moreover, individuals with mutations in the NALP3 gene were shown to secrete more IL-18, in addition to secreting elevated levels of IL-1β, indicating that both IL-1β and IL-18 contribute to systemic inflammation. Additional attention must also be paid to the role played by reactive oxygen species [63] produced by NLRP3/NALP3 which is a required second messenger for inflammasome activation as well as potentially exploiting a recent finding that a family of mammalian NIMA-related kinases, in particular the NEK7 isoform, serves as a NLRP3-binding protein acting downstream of potassium efflux that regulates NLRP3 oligomerization and activation [64]. Thus, NEK7 could become a future target for limiting NLRP3 inflammasome assembly and activation [64].

## Abbreviation

ACS	adaptor protein/pyrin domain of apoptosis/speck-like protein containing a caspase-recruitment domain
ATP-gated P2X7 receptor	adenosine triphosphate-gated P2X7 receptor
CAPS	cryopyrin-associated periodic syndrome
CARD	caspase-recruitment domain
CARDINAL	caspase-recruitment domain-8
CD	cluster of differentiation
COX-2	cyclooxygenase-2
CRP	C-reactive protein
c-Rel	reticuloendotheliosis viral oncogene-like homolog
ESR	erythrocyte sedimentation rate
FCAS	familial cold autoinflammatory syndrome
FMF	Familial Mediterranean fever
GST-tagged	glutathione-S-transferase-tagged
HIDS	hyperimmunoglobulinemia D syndrome
IL-1β, IL-6	interleukin-1β, interleukin-6, interleukin-8, interleukin-12, interleukin-18
iNOS	inducible nitric oxide synthase
LPS	lipopolysaccharide
MA	mevalonic aciduria
MVA	mevalonic aciduria
MVK	mevalonate kinase
MWS	Muckle-Wells syndrome
NACHT	neuronal apoptosis protein/class 2 transcription factor/heterokaryon incompatibility/telomerase-associated protein-1
NALP3	nucleotide-binding domain/leucine-rich repeat/pyrin-domain containing-3
NLRP3	Nod-like receptor protein 3
NF-κB	nuclear factor-kappa B
NBC	B30.2 domain-deleted pyrin
NK	natural killer
NLR	nucleotide-binding, oligomerization domain receptor
NIMA	Aspergillus nidulans ‘never in mitosis A’
NEK7	NIMA-related kinase-7
NO	nitric oxide
PAF	platelet activating factor
PAMPS	pathogen-associated molecular patterns
PAPA	pyogenic arthritis, pyoderma gangrenosum and acne
PGE_2_	prostaglandin E2
PSTPIP1	proline-serine-threonine phosphatase interacting protein 1
PYD	pyrin domain
PYCARD	pyrin/caspase-recruitment domain
SAA	serum amyloid A
TNF-α	tumor necrosis factor-α
TNFRSF1A	tumor necrosis factor receptor superfamily, member 1A
NOMID/CINCA	neonatal onset multi-systemic inflammatory disease/chronic infantile neurological cutaneous articular syndrome
TRAPS	tumor necrosis factor receptor-associated periodic syndrome
